# Human cell-expressed tag-free rhMFG-E8 as an effective radiation mitigator

**DOI:** 10.1038/s41598-023-49499-y

**Published:** 2023-12-13

**Authors:** Wayne Chaung, Gaifeng Ma, Asha Jacob, Max Brenner, Ping Wang

**Affiliations:** 1 TheraSource LLC, 350 Community Drive, Manhasset, NY USA; 2 Center for Immunology and Inflammation, Feinstein Institutes for Medical Research, 350 Community Drive, Manhasset, NY 11030 USA; 3 Departments of Surgery and Molecular Medicine, Zucker School of Medicine, Hempstead, NY USA

**Keywords:** Biotechnology, Drug discovery, Molecular biology, Gastroenterology, Medical research

## Abstract

Human milk fat globule epidermal growth factor-factor VIII (MFG-E8) functions as a bridging molecule to promote the removal of dying cells by professional phagocytes. *E. coli*-expressed histidine-tagged recombinant human MFG-E8 (rhMFG-E8) is protective in various disease conditions. However, due to improper recombinant protein glycosylation, misfolding and the possibility of antigenicity, *E. coli*-expressed histidine-tagged rhMFG-E8 is unsuitable for human therapy. Therefore, we hypothesize that human cell-expressed, tag-free rhMFG-E8 will have suitable structural and functional properties to be developed as a safe and effective novel biologic to treat inflammatory diseases including radiation injury. We produced a new tag-free rhMFG-E8 protein by cloning the human MFG-E8 full-length coding sequence without any fusion tag into a mammalian vector and expressed it in HEK293-derived cells. The construct includes the leader sequence of cystatin S to maximize secretion of rhMFG-E8 into the culture medium. After purification and confirmation of the protein identity, we first evaluated its biological activity in vitro. We then determined its efficacy in vivo utilizing an experimental rodent model of radiation injury, i.e., partial body irradiation (PBI). HEK293 cell supernatant containing tag-free rhMFG-E8 protein was concentrated, purified, and rhMFG-E8 was verified by SDS-PAGE with the standard human MFG-E8 loaded as control and, mass spectrometry followed by analysis using MASCOT for peptide mass fingerprint. The biological activity of human cell-expressed tag-free rhMFG-E8 was superior to that of *E. coli*-expressed His-tagged rhMFG-E8. Toxicity, stability, and pharmacokinetic studies indicate that tag-free rhMFG-E8 is safe, highly stable after lyophilization and long-term storage, and with a terminal elimination half-life in circulation of at least 1.45 h. In the 15 Gy PBI model, a dose-dependent improvement of the 30-day survival rate was observed after tag-free rhMFG-E8 treatment with a 30-day survival of 89%, which was significantly higher than the 25% survival in the vehicle group. The dose modification factor (DMF) of tag-free rhMFG-E8 calculated using probit analysis was 1.058. Tag-free rhMFG-E8 also attenuated gastrointestinal damage after PBI suggesting it as a potential therapeutic candidate for a medical countermeasure for radiation injury. Our new human cell-expressed tag-free rhMFG-E8 has proper structural and functional properties to be further developed as a safe and effective therapy to treat victims of severe acute radiation injury.

## Introduction

Human milk fat globule epidermal growth factor-factor VIII (MFG-E8) is a 387-amino acid glycoprotein also known as lactadherin. It was first identified as an indispensable and major component of the milk fat globule in the lactating mammary glands^1^. Since its discovery, MFG-E8 has shown to be expressed in various cell types including mammary epithelial cells, keratinocytes, dendritic cells, glial cells, splenocytes, monocytes, peritoneal macrophages, and lamina propria macrophages in the small intestine and the colon^2–4^. Human MFG-E8 contains one epidermal growth factor (EGF)-like repeat at the N-terminal and two discoidin domains at the C-terminal. MFG-E8 received its name based on the sequence similarity of the EGF-like repeat to those of EGF and of the discoidin domains to those of the blood coagulation factor V/VIII segments^5^. Its N-terminal EGF-like domain contains an RGD (Arg-Gly-Asp) motif that binds α_V_β_3_ and α_V_β_5_ integrins on the surface of macrophages while the C-terminal, factor VIII-like discoidin domain binds phosphatidylserine (PS) on the surface of apoptotic and other dying cells. Thus MFG-E8 functions as a bridging molecule to promote removal of unwanted cells by professional phagocytes^5,6^. MFG-E8 deficient mice display inflammation and autoimmunity and have glomerulonephritis due to defects in apoptotic cell engulfment by phagocytes^7^. Since MFG-E8 promotes clearance of apoptotic cells, we reasoned that exogenous administration of MFG-E8 could prevent the exaggerated inflammatory responses seen in acute inflammation such as sepsis^8,9^. As such, we produced polyhistidine (His)-tagged recombinant human (rh)MFG-E8 using an *E. coli* expression system and conducted proof-of-concept studies in various experimental rodent models of acute inflammation^10–15^.

Glycosylation is thought to enhance the amphipathic properties of MFG-E8 afforded by its membrane binding discoidin domains and its hydrophilic N‐terminal tail^16^. However, *E. coli* lacks the cellular machinery to adequately conduct many critical post-translational modifications, including glycosylation and phosphorylation^17^. Consequently, recombinant protein misfolding and aggregation in the form of inclusion bodies are common in prokaryotic expression systems^18^. Moreover, His-tagging increases the immunogenicity of recombinant proteins^19^. Together, inadequate glycosylation, protein misfolding, and potential immunogenicity render *E. coli*-expressed His-tagged rhMFG-E8 unsuitable to be used therapeutically in humans. To circumvent these limitations, we set out to produce a tag-free, normally glycosylated rhMFG-E8 to be further developed as a biologic treatment for acute inflammation. Towards this objective, we cloned the full-length coding sequence of human MFG-E8 devoid of any fusion protein tag into a mammalian vector and expressed in a human cell line. After purification and confirmation of the protein identity, we evaluated its safety and biological activity in vitro and in vivo. To test its biological activity in vitro, we used a novel PS binding assay and a cell adhesion assay. Toxicity, stability, and pharmacokinetics were assessed in vivo.

To test the efficacy in vivo, we utilized a rodent model of acute radiation syndrome (ARS). ARS is a severe form of radiation injury that occurs after non-medical exposure to high doses of penetrating radiation within a relatively short period of time^20–22^. ARS occurs after total-body irradiation (TBI) or significant partial body irradiation (PBI) typically at a dose of > 1 Gy. Exposure to elevated doses of radiation may happen after a natural disaster or an accident in a nuclear facility, or the detonation of a radioactive device or nuclear bomb due to war or international terrorism. ARS can result in three overlapping and progressively severe syndromes. The stem and progenitor cells that regenerate blood cells, and the mucosal lining of the digestive system have elevated mitotic rates, which make them particularly susceptible to damage by ionizing radiation^22^. Exposures to above > 2–3 Gy cause the hematopoietic syndrome (H-ARS) characterized by neutropenia resulting in life-threatening infections along with thrombocytopenia leading to spontaneous bleeding. Exposures to above 5–12 Gy cause the gastrointestinal syndrome (GI-ARS) characterized by vomiting, severe diarrhea, structural damage to the gut, and translocation of bacteria from the gut to the circulation. The neurovascular syndrome (NV-ARS) happens after the exposure to extremely elevated doses of radiation (10–20 Gy) and invariably progresses to death within days^23^. Several growth factors have been FDA-approved as medical countermeasures (MCMs) to treat patients with H-ARS: Neupogen (filgrastim), Neulasta (pegfilgrastim), Leukine (sargramostim), NPLATE (romiplostim), and Udenyca (pegfilgrastim-cbqv). No FDA-approved radiation mitigators, however, appear to be effective against GI-ARS. Therefore, there is an urgent unmet need for an effective MCM for GI-ARS. In a rodent model of TBI, we have previously shown that MFG-E8 levels in the circulation and its mRNA expression in the intestine were significantly reduced after TBI, and that reconstitution of the MFG-E8 deficit with exogenous *E. coli*-expressed His-tagged rhMFG-E8 preserved intestinal histological architecture leading to improved survival after TBI^11^. These results strongly support developing rhMFG-E8 as a biologic mitigator for GI-ARS.

## Materials and methods

### Tag-free rhMFG-E8 expression and purification from human cells

The full-length coding sequence of human MFG-E8 (NM_005928.2; Leu24-Cys387), with no fusion tag but addition of the 23 amino acid leader sequence of cystatin S to maximize secretion, was cloned into a mammalian vector and expressed in Expi293F™ cells (Life Technologies), which are derived from the human embryonic kidney (HEK) 293 cell line. The human cell-expressed tag-free rhMFG-E8 protein secreted into the culture medium was purified by cation exchange using fast protein liquid chromatography (FPLC) and eluted protein fractions were monitored at 280-nm absorbance and collected. The elution fractions of the target protein were run on SDS-PAGE with the commercially available His-tagged rhMFG-E8 as a positive control, detected by Coomassie blue stain for purity confirmation and Western blotting using anti-MFG-E8 antibody (R&D) for specificity. The gel band containing the target protein was then digested by trypsin, which cleaves peptides on the C-terminal side of the lysine and arginine amino acid residues, and subjected to MALDI-TOF mass spectrum and the m/z (mass/charge) ratios of the peaks obtained were searched in SWISSPROT database with MASCOT Server for peptide mass fingerprint analysis^24^.

### Biological activity assays for human cell-expressed tag-free rhMFG-E8

We developed two quality control assays and one of which is to verify the tag-free rhMFG-E8’s ability to bind to PS on the surface of dying cells (PS binding assay) and another is the tag-free rhMFG-E8 to bind to α_V_β_3_ integrin on the membrane of phagocytes (cell adhesion assay). Briefly, for PS binding assay, we first coated 96-well plate with 100 µl of 3 µg L-a-phosphatidylserine/mL methanol (PS, Avanti Polar Lipids, Inc.) in a laminar flow hood overnight. After washing the plate 3 times with PBS containing 0.05% Tween-20 (PBS-T), the wells were blocked with 2% BSA in PBS for 2 h. After washing again, 100 µl each of serially diluted purified tag-free rhMFG-E8 protein or commercially available mammalian cell-expressed His-tagged rhMFG-E8 (R&D Systems, cat: 2767-MF) was added to PS-coated wells and the plate was incubated for 1 h at room temperature on a rotator. After the washes, 100 µl each of 0.5 µg/ml human MFG-E8 primary antibody (R&D Systems, cat: MAB27671) was added to each well and the plate incubated for 1 h. After the wash, 100 µl each of 1:10,000 dilution of the secondary antibody, goat anti-mouse IgG-HRP (Southern Biotech, cat: 1012–05) was added to each well and incubated for 1 h. After washing, 100 µl TMB substrate reagent (BD, Cat: 555214) was added to each well and waited for 30 min. Then, 50 µL 2N H_2_SO_4_ stop solution was added to each well. The plate was read at 450 nm with a plate reader (SynergyNeo2, BioTek). The OD vs. concentration was plotted to detect the amount of rhMFG-E8 that remained bound to the plate and the slope was calculated. For the cell adhesion assay, we determined the ability of rhMFG-E8 to bind α_V_β_3_ integrin. Fluorescently labelled mouse vascular endothelial cells (SVEC4–10, ATCC), characterized by high surface expression of α_V_β_3_ integrin, were added to wells pre-coated with different concentrations of rhMFG-E8. After washes, the plate was read using a fluorescence plate reader (SynergyNeo2, BioTek). The percentage of cells that remained bound to the plate was quantified.

### Experimental animals

Sprague Dawley rats (275–350 g) were obtained from Charles River Laboratories. C57BL6 mice were purchased from Jackson Laboratory. Both were housed in a temperature-controlled room with a 12-h light/dark cycle and fed a standard Purina mouse chow diet. Both rats and mice were acclimated to the environment for 5 to 7 days. Age-matched (12–16 weeks for rats and 8–12 weeks for mice) healthy animals were used as controls for the experiments. All experiments involving live animals were approved by the Institutional Animal Care and Use Committee of the Feinstein Institutes for Medical Research and were performed in accordance with the National Institutes of Health and the Guide for the Care and Use of Laboratory Animals. The study was conducted and reported in accordance with ARRIVE guidelines 2.0.

### Toxicity and stability assessment of human cell-expressed tag-free rhMFG-E8

Non-anesthetized adult male and female healthy mice received a single subcutaneous (*sc*) injection of tag-free rhMFG-E8 at various doses and the mice were monitored daily for 28 days and body weight recorded. Additional mice, untreated or treated with a single *sc* injection of up to 2.0 mg/kg BW tag-free rhMFG-E8, were followed for 1, 7, or 28 days, when blood was collected for further analysis. To assess the stability of the protein after long term storage, tag-free rhMFG-E8 was lyophilized and stored at – 20 °C. Every four months over a total period of 2 years, the lyophilized protein was reconstituted with pure water and subjected to PS binding and cell adhesion assays to evaluate the stability of its biological activity. MFG-E8 protein purchased from R&D was used each time as a control for assay.

### Determination of pharmacokinetic properties of human cell-expressed tag-free rhMFG-E8

To allow for repeated collection of sufficient amounts of blood, pharmacokinetic studies were performed in Sprague Dawley rats. Rats were intravenously injected with 50 µg of tag-free glycosylated rhMFG-E8. Blood samples (100 μl per time point) were collected at 0, 3, 6, 9, 12 and 15 min, then every 15 min for the first 60 min, and then every hour for additional 11 h. Equal volume (100 µl) of blood withdrawn from the rat was replenished with normal saline. The total volume of blood collected (1.6 ml) was approximately 8% of the total body blood volume (~ 20 ml/rat). The levels of the tag-free rhMFG-E8 from rat blood samples were measured by Human MFG-E8 Quantikine ELISA kit (R&D Systems, Cat: MFGE80). There is no cross-reaction between human and rat MFG-E8 in this kit. Serum rhMFG-E8 concentration vs. time were plotted and slopes of the distribution (α) and elimination (β) phases were calculated. The plasma values obtained from 0 to 0.5 h was designated as the α phase and those obtained from 2 to 6 h time points as the β phase. The plasma data after intravenous bolus input were re-calculated using a non-compartmental analysis with a log-linear trapezoidal method in the PKSolver^25^.

### Mouse model of PBI and treatment with human cell-expressed tag-free rhMFG-E8

Non-anesthetized adult male and female mice were exposed to a PBI dose of 15 Gy in an X-Ray irradiator (model: X-RAD 320, Pxi, North Branford, CT) at a rate of approximately 1 Gy/min at 320 kV, 12.5 mA, 50 cm SSD. We first exposed the mice to different doses of X-ray using PBI model and determined 15 Gy as the optimal dose to ensure high mortality rate in C57BL/6 J mice. During irradiation, mice were briefly restrained and placed in a fitted container with the hind extremities (fibula, tibia and feet) were shielded using lead tubes, thereby protecting approximately 5% of the bone barrow. After PBI, the mice were returned to their cages. At 24 h later, mice were *sc* injected daily for 6 days with normal saline (vehicle) or tag-free rhMFG-E8 (treatment) at various doses. The mice were monitored daily for 30 days, and survival rate recorded.

### Determination of dose modification factor (DMF) of human cell-expressed tag-free rhMFG-E8

Mice were exposed to PBI at doses of 13, 13.5, 14, 14.5, 15 and 16 Gy. At 24 h later, they were injected *sc* with either vehicle or 1 µg/kg BW tag-free rhMFG-E8 daily for 6 consecutive days, monitored daily for 30 days and survival recorded. The survival data were plotted as percent mortality vs. PBI dose and DMF was calculated using probit analysis in Microsoft Excel 365.

### Assessment of gut damage after PBI and treatment with human cell-expressed tag-free rhMFG-E8

Mice exposed to 14.5 Gy PBI were injected *sc* with 1 µg/kg BW tag-free rhMFG-E8 for 3 consecutive days starting at 24 h after PBI. They were then euthanized, and blood and jejunal segments of the gut were harvested for further analysis. The serum levels of citrulline from PBI samples were measured using commercially available enzyme-linked immunosorbent assay (ELISA) kits (Mouse Citrulline ELISA Kit, MyBioSource, cat # MBS2602136). Plasma citrulline has been identified as a biomarker of radiation-induced gut damage^26^. Jejunal segments were paraffin-embedded, sectioned, and stained with H&E. Gut histological damage score for PBI samples was quantified using RIIMS, a detailed 7-category scoring system previously described^11^.

### Statistical analysis

Results are expressed as mean ± SEM and compared by using one-way analysis of variance (ANOVA) and Student Newman-Keul’s (SNK) post hoc analysis. Survival rates were analyzed by the Kaplan–Meier estimation and compared using log-rank test. Differences in values were considered significant if P < 0.05. Data analyses were conducted using GraphPad statistical program and software (GraphPad).

### Ethics approval and consent to participate

All experiments involving live animals were approved by the Institutional Animal Care and Use Committee of the Feinstein Institutes for Medical Research and were performed in accordance with the National Institutes of Health and the Guide for the Care and Use of Laboratory Animals.

## Results

### Synthesis and purification of human cell-expressed tag-free rhMFG-E8

**Figure 1:**
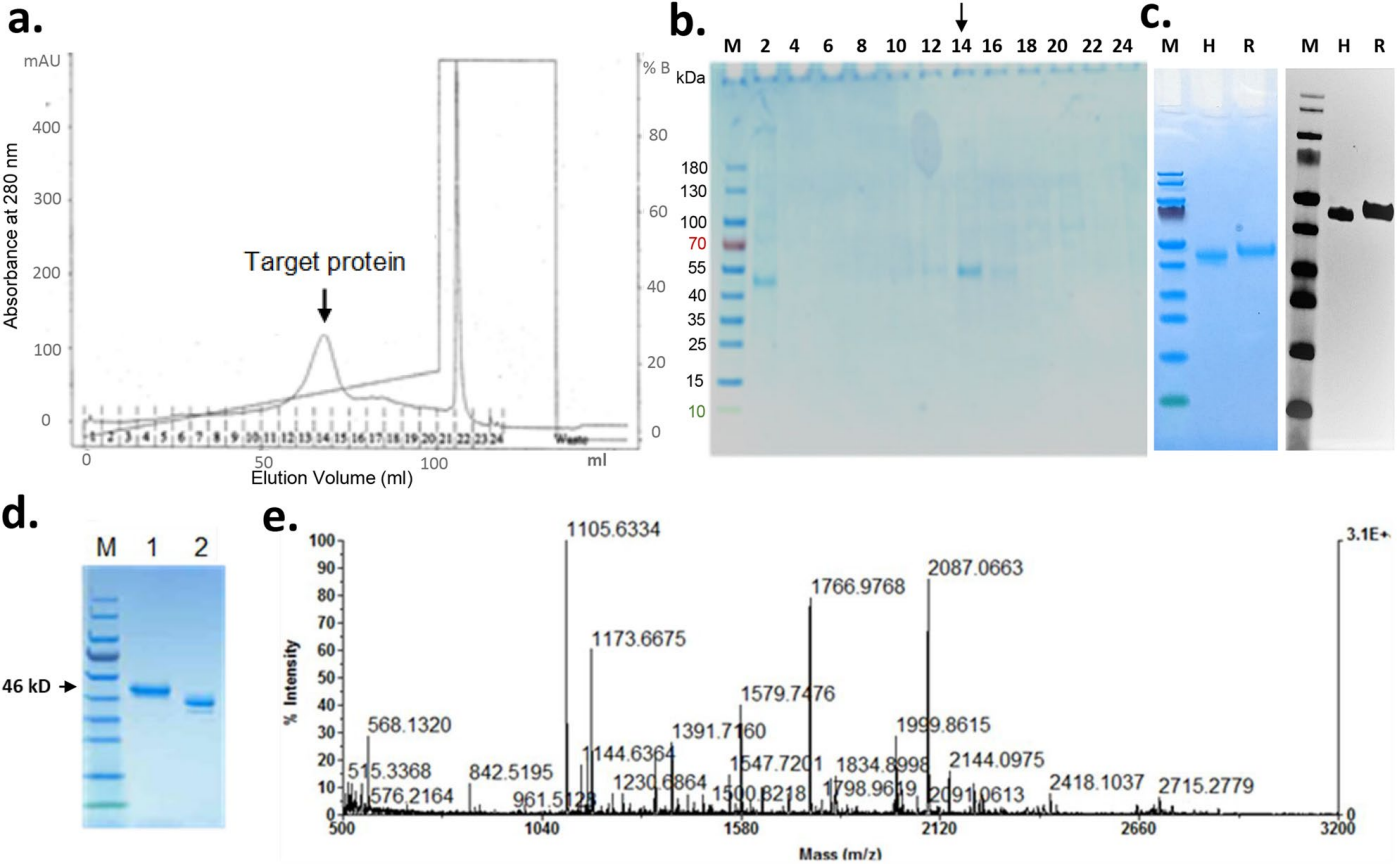
Expression and purification of tag-free rhMFG-E8. Tag-free rhMFG-E8 was expressed in human cells and purified. (**a**) The eluted protein’s 280-nm absorbance was monitored using FPLC and the target protein fraction (arrow) identified. (**b**) Eluted protein fractions from FPLC were confirmed by polyacrylamide gel electrophoresis, and (**c**) NuPAGE 4–12% Bis–Tris gel with tag free rhMFG-E8 (H) was verified for its authenticity using commercially available His-tagged rhMFG-E8 (R) stained with Coomassie (left panel) and Western blotted using anti-MFG-E8 antibody (right panel), (**d**) fraction #14 was resolved separately in a NuPAGE 4–12% Bis–Tris gel under reducing (Lane 1) and non-reducing (Lane 2) conditions. (**e**) The purified protein was trypsinized, peaks separated with MALDI-TOF mass spectrum and the m/z (mass/charge) ratios of the peaks obtained were searched in MASCOT Server for peptide mass fingerprint analysis. The analysis confirmed the target protein as lactadherin or human MFG-E8.

Tag-free rhMFG-E8 with 23 aa leader sequence of cystatin S was cloned into a mammalian expression vector, expressed in human HEK cells, and purified with cation exchange using FPLC. The eluted protein fractions were monitored with 280-nm absorbance and a target protein peak was identified (Fig. 1a) and collected. After optimization of the expression procedure, the concentration of the tag-free glycosylated rhMFG-E8 was high enough to be detected directly by SDS-PAGE and fraction #14 contained the majority of the expressed rhMFG-E8 protein, with a concentration of 0.33 µg/µl (Fig. 1b). A separate SDS-PAGE gel with the commercially available human MFG-E8 (His-tagged rhMFG-E8; R&D) as control and the tag-free rhMFG-E8 were electrophoresed; one gel was stained with Coomassie and the second gel was Western blotted using anti-MFG-E8 antibody. As expected, the His-tagged rhMFG-E8 showed a slight increase (0.8 kDa) in molecular weight due to the addition of six histidines as a fusion protein but both proteins were recognized by an anti-MFG-E8 antibody (Fig. 1c). The purity of the fraction #14 was further verified by SDS-PAGE under reducing and non-reducing conditions (Fig. 1d), and the excised gel band protein was digested with trypsin and sequenced using mass spectrometry (Fig. 1e). The m/z (mass/charge) ratios of the peaks obtained from the mass spectrum data were searched using the SWISSPROT database in MASCOT Server for peptide mass fingerprint^24^. The search confirmed the target protein as lactadherin/human MFG-E8. The purified tag-free rhMFG-E8 using human cell expression system had an endotoxin level below 0.02 EU/µg (data not shown), about two orders of magnitude lower than the industry standard (< 1 EU/µg).

### Human cell-expressed tag-free rhMFG-E8 has high and stable biological activity

**Figure 2:**
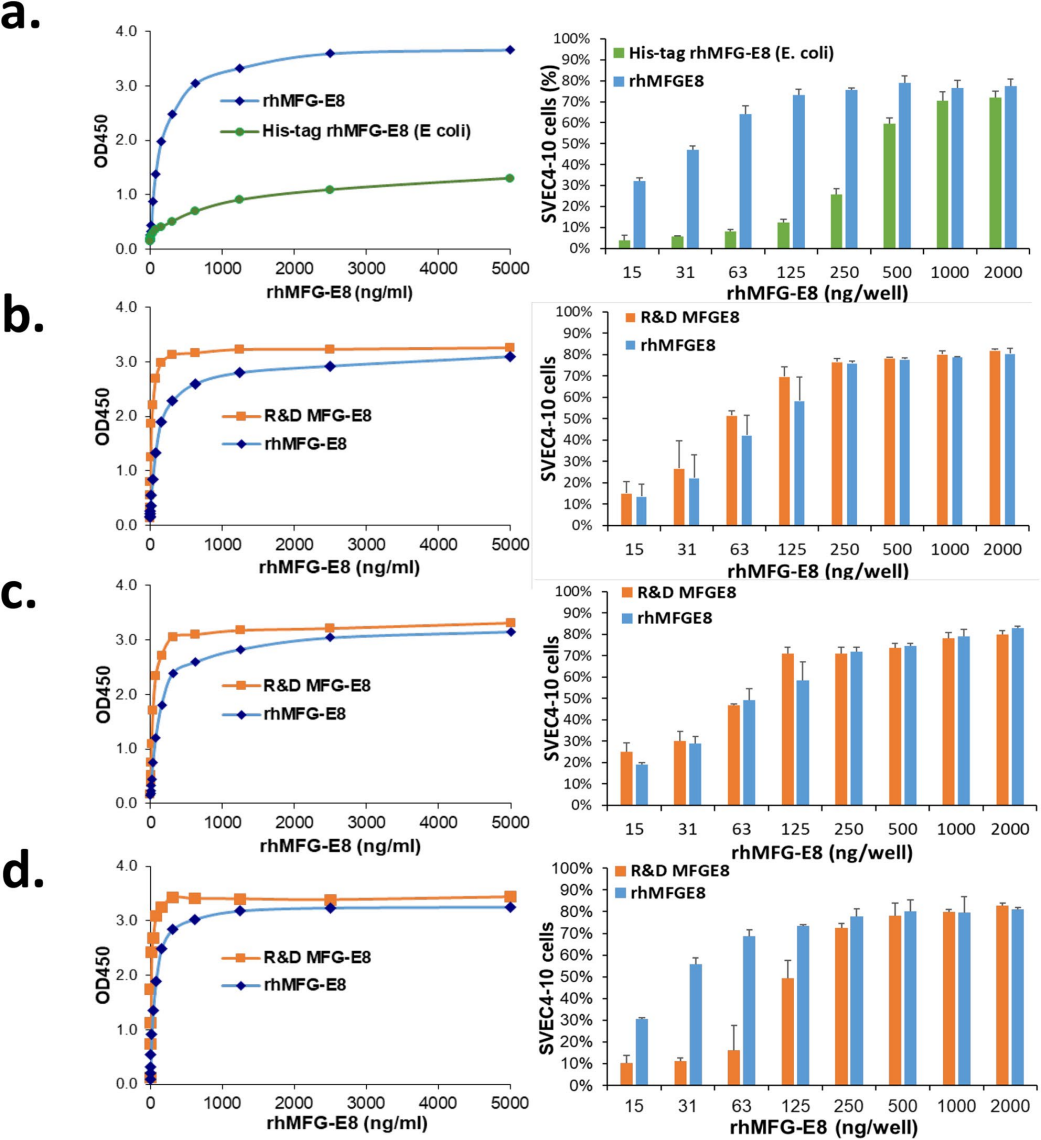
(**a**) Tag-free rhMFG-E8 is superior to *E coli* expressed His-tagged rhMFG-E8. PS binding (left panel) and SVEC4-10 cells adhesion assay (right panel) using tag-free and His-tagged rhMFG-E8. (**b**) Tag-free rhMFG-E8 has high biological activity. PS binding (left panel) and SVEC4-10 cell adhesion assay (right panel) of commercial MFG-E8 (R&D MFG-E8) and tag-free rhMFG-E8 (rhMFG-E8). (**c,d**) *Tag-free rhMFG-E8 is highly stable.* PS binding and cell adhesion assays performed at (**c**) twelve months, (**d**) twenty-four months after storage of the tag-free rhMFG-E8 at − 20 °C.

The biological activity of the tag-free rhMFG-E8 was assessed using two methods. We independently verified its ability to bind to both PS and α_V_β_3_ integrin. To determine the PS binding ability, various concentrations of tag-free rhMFG-E8, *E. coli*-expressed His-tagged rhMFG-E8 or commercially available rhMFG-E8 (R&D) were added to PS-coated wells and the amount of rhMFG-E8 that remained bound to the plate was assessed using a chromogenic immunoassay with anti-MFG-E8 antibodies. The PS binding activity of human cell-expressed tag-free rhMFG-E8 was significantly increased than that of *E. coli*-expressed His-tagged rhMFG-E8 (Fig. 2a, left panel). We compared our human cell-expressed tag-free rhMFG-E8 against commercially available rhMFG-E8 (R&D), which is also mammalian cell-expressed but unsuitable to be used therapeutically because it is His-tagged. As indicated by a steep linear phase slope, tag-free rhMFG-E8 had very comparable PS-binding activity to that of His-tagged commercially available rhMFG-E8 (Fig. 2b, left panel). To determine the ability of rhMFG-E8 to bind to α_V_β_3_ integrin, fluorescently labelled mouse vascular endothelial cells SVEC4-10, which express α_V_β_3_ integrin on their surface, were added to wells pre-coated with different concentrations of various sources of rhMFG-E8. After washing, the percentage of fluorescent cells that remained bound to the plate was quantified. Echoing our PS binding results, the cell adhesion binding activity of human cell-expressed tag-free rhMFG-E8 was also markedly higher than that of *E. coli*-expressed His-tagged rhMFG-E8 (Fig. 2a, right panel). Tag-free rhMFG-E8 also bound SVEC4-10 cells more strongly and at much lower concentrations than the His-tagged commercially available rhMFG-E8 (Fig. 2b, right panel). These results demonstrate that the biological activity of tag-free rhMFG-E8 is far superior to that of *E. coli*-expressed His-tagged rhMFG-E8, and comparable (if not better) to His-tagged commercially available rhMFG-E8. In terms of stability of lyophilized, stored, and then reconstituted tag-free rhMFG-E8, we conducted a total of 12 independent sets of PS binding and cell adhesion assays over a 2-year period. Our results indicated that both PS binding ability and SVEC4-10 cell binding capability of the stored tag-free rhMFG-E8 were highly similar to those of His-tagged commercially available MFG-E8, which benefits from R&D’s excellent industrial quality control assessment (Fig. 2c,d).

### Human cell-expressed tag-free rhMFG-E8 has improved and stable biologic activity, is safe, and has adequate pharmacokinetic properties

**Figure 3:**
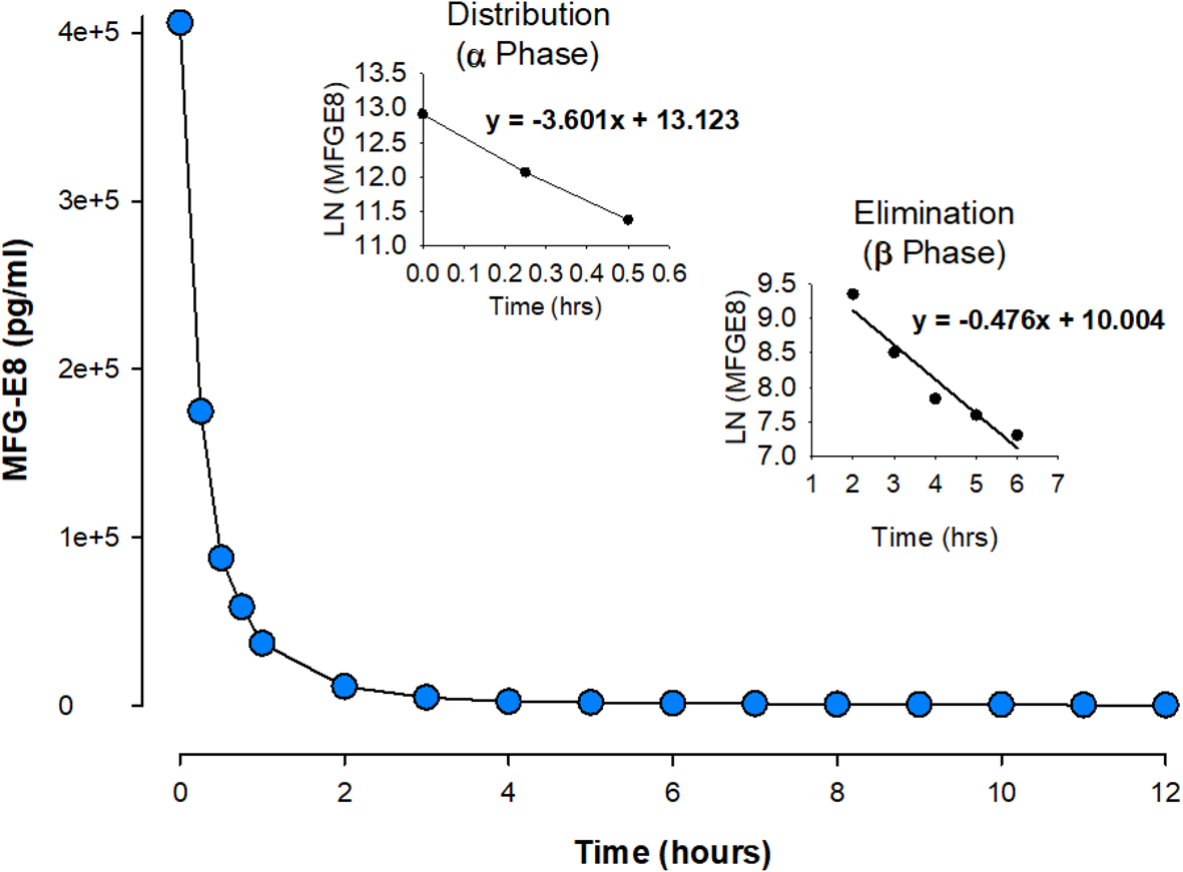
Tag-free rhMFG-E8 has suitable pharmacokinetics in healthy animals. Rats were *iv* injected with tag-free MFG-E8 and blood samples collected at different timepoints. The levels of the tag-free rhMFG-E8 were measured using human MFG-E8 Quantikine ELISA kit. The data were plotted as serum rhMFG-E8 concentration against time points to determine the t_1/2_. The α phase represents the distribution phase and the β phase represents the elimination phase. We designated the α phase as 0 to 0.5 h and the β phase as 2 to 6 h time points.

To screen for major toxicity, we injected naïve mice with tag-free rhMFG-E8 and analyzed the sera collected at 1-, 7-, and 28-days post-injection for the organ injury biomarkers AST, ALT, LDH, creatinine, BUN, and for the pro-inflammatory cytokines TNF-α and IL-6. All biomarker and cytokine measurements were within normal levels suggesting tag-free rhMFG-E8 treatment of healthy mice causes neither acute nor chronic toxicity (Table 1). We attempted to determine the maximum tolerated dose, but no deaths were observed in mice treated with doses of up to 2 mg/kg BW tag-free rhMFG-E8 and they didn’t undergo any significant changes in body weight. These results showed that very high doses of tag-free rhMFG-E8 (up to 2000-fold higher than the highest dose used in this study) produced no measurable toxicity in mice. To assess pharmacokinetics, wild type Sprague Dawley rats were injected with a bolus dose of 50 µg tag-free rhMFG-E8 and serial blood samples collected at different time points were analyzed for the concentration of the exogenous tag-free rhMFG-E8 using an ELISA kit that does not recognize rat MFG-E8. The terminal elimination half-life (t_1/2_) of the tag-free rhMFG-E8 was calculated for each of 4 independent experiments and averaged. Tag-free rhMFG-E8’s average α phase (distribution) t_1/2_ was 11.55 min, and its average β phase (elimination) t_1/2_ was 1.45 h (Fig. 3). The PK analysis of the plasma data after intravenous bolus input with a non-compartmental analysis and log-linear trapezoidal method using PKsolver were also calculated. By using the plasma values obtained from 2 to 6 h which we designated as the β phase, we obtained a t_1/2_ value of 1.45 h which was identical to what was observed with the prior analysis. However, when the time was extended to 12 h instead of 6 h, the t_1/2_ increased to 2.33 h. Nevertheless, these data indicate that the β phase t_1/2_ of the tag-free rhMFG-E8 is calculated as at least 1.45 h.

**Table 1:** Tag-free rhMFG-E8 does not cause acute or chronic toxicity in healthy mice.

Assessment	Baseline	rhMFG-E8 (2 mg/kg), Day 0		
		Day 1	Day 7	Day 28
AST (IU/L)	21.2 ± 4.8	29.3 ± 10.6	13.8 ± 4.0	18.8 ± 0.3
ALT (IU/L)	3.7 ± 1.4	2.7 ± 2.1	2.4 ± 1.3	3.3 ± 1.6
LDH (U/L)	19.0 ± 11.3	52.9 ± 45.9	10.8 ± 5.1	27.6 ± 11.1
Creatinine (mg/dL)	0.3 ± 0.1	0.3 ± 0.1	0.4 ± 0.1	0.4 ± 0.02
BUN (mg/dL)	20.6 ± 2.3	21.8 ± 2.6	20.7 ± 2.2	24.7 ± 0.5
TNF-α (pg/ml)	1.2 ± 0.3	2.0 ± 0.5	1.7 ± 0.3	2.0 ± 0.1
IL-6 (pg/ml)	4.3 ± 0.8	11.4 ± 2.3	6.1 ± 1.1	8.0 ± 1.8

These results collectively demonstrated that human cell-expressed tag-free rhMFG-E8 has superior biological activity, is highly stable, safe, and has suitable pharmacokinetic properties to be used as a novel biologic treatment.

### Human cell-expressed tag-free rhMFG-E8 showed dose-related improvement in survival after PBI

**Figure 4:**
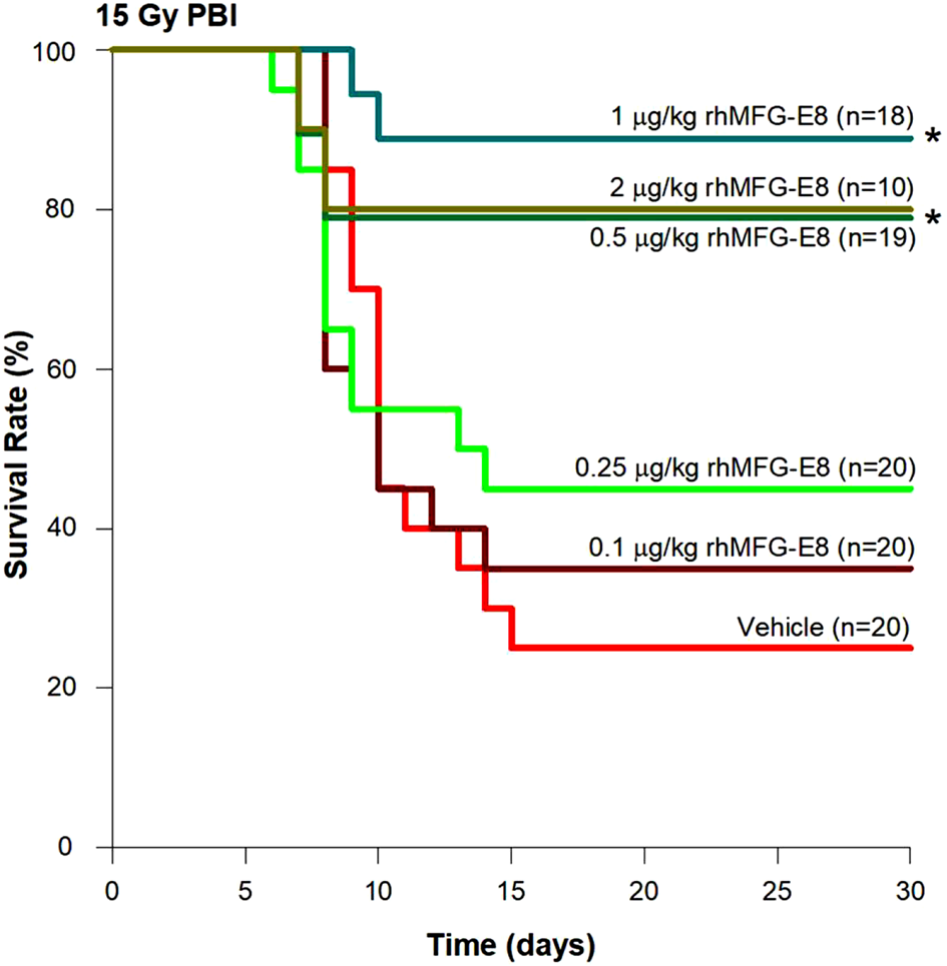
Dose response relationship of tag-free rhMFG-E8. Mice were exposed to PBI at 15 Gy and received sc injections of normal saline (Vehicle) or the indicated various amounts of tag-free rhMFG-E8 (rhMFG-E8) for 6 days, starting at 24 h after PBI. The 30-day survival was recorded. *P < 0.05 vs. Vehicle.

The PBI model was then used to evaluate the effect of tag-free rhMFG-E8 on GI-ARS. We subjected mice to 15 Gy PBI and, at 24 h after irradiation, treated them with either vehicle or varying doses of tag-free rhMFG-E8 to determine the optimal dose of rhMFG-E8 to improve survival. We observed a dose-dependent improvement of the 30-day survival rate with the treatment group (Fig. 4). The highest survival rate reached 89% at 1 µg/kg BW tag-free rhMFG-E8 group, which was significantly higher than the vehicle group at 25%. When administered tag-free rhMFG-E8 at 2 µg/kg BW, it did not show further improvement in survival rate in comparison to the 1 µg/kg BW group. Therefore, 1 µg/kg BW tag-free rhMFG-E8 was chosen as the optimal dose to conduct subsequent studies.

### Human cell-expressed tag-free rhMFG-E8 produced an effective dose modification factor (DMF) after PBI

**Figure 5:**
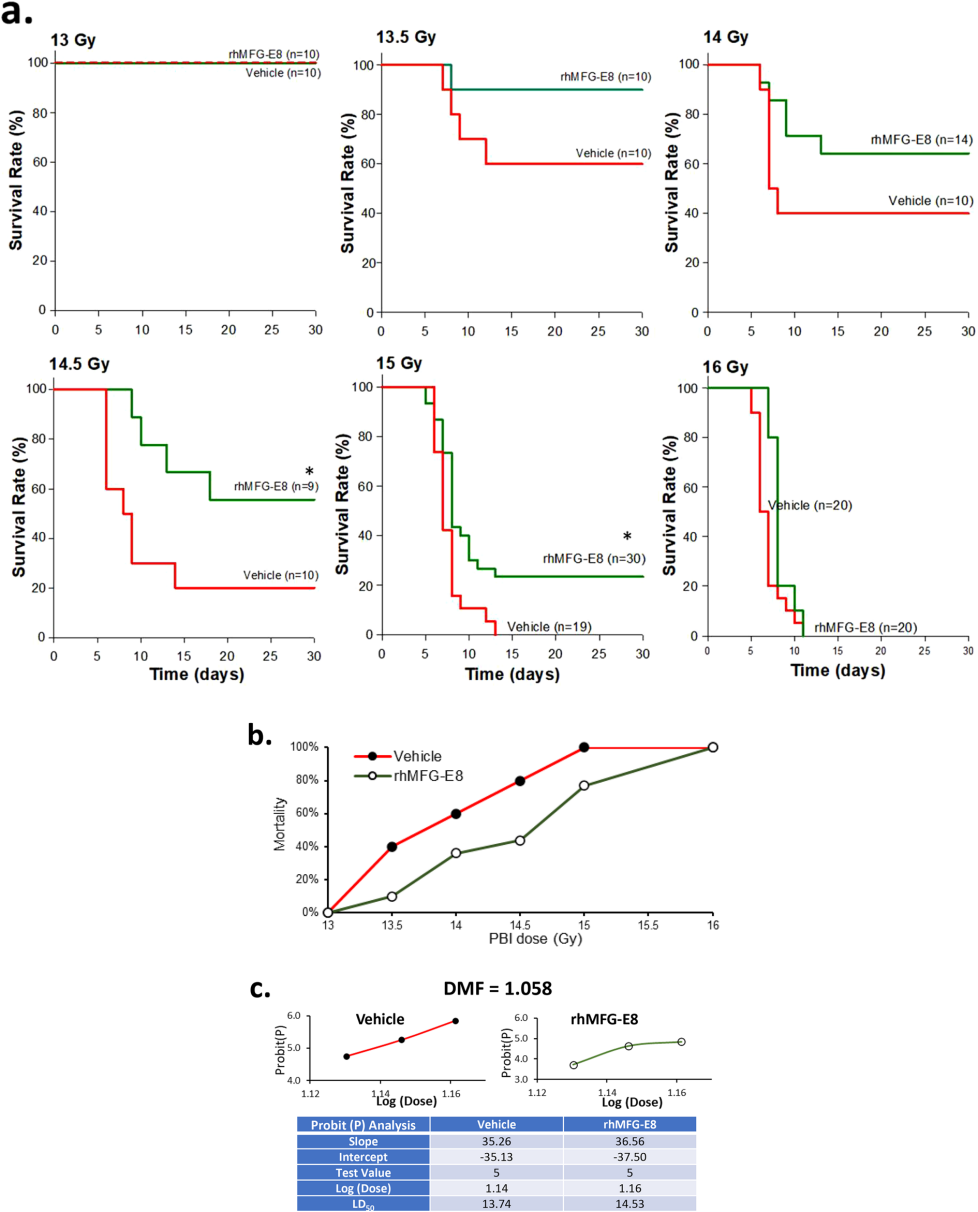
Determination of the dose modification factor (DMF) in PBI mice. (**a**) Mice were exposed to PBI at the indicated doses and injected sc with normal saline (Vehicle) or 1 ug/kg tag-free rhMFG-E8 (rhMFG-E8) for 6 days starting at 24 h post-PBI, observed for 30 days and the survival recorded. (**b**) Percent mortality was plotted against each irradiation dose for the 30-day survival. (**c**) Probit (P) analysis and DMF calculation of the data plotted in (**b**). *P < 0.05 versus vehicle.

The DMF, also called dose reduction factor, has been considered the best effectiveness index for radiation MCM and is defined as a ratio between the dose of irradiation required for a given effect of a drug-treated group and that of the vehicle-treated group. DMF is essential to label a drug as a radiation MCM. To determine the DMF, although not ideal, we used the “same-dose of radiation” comparisons. Based on previous experience with radiation studies, doses with 0.5 Gy increments were chosen to capture the LD_50/30_ dose for the vehicle group. Using this approach, mice were irradiated at 13, 13.5, 14, 14.5, 15 and 16 Gy PBI and 24 h later, they were treated with either normal saline (vehicle) or 1 µg/kg BW tag-free rhMFG-E8 *sc* for 6-days. The 30-day survival rates were recorded (Fig. 5a). Vehicle and rhMFG-E8 treated mice had no mortality after 13 Gy PBI, and 100% mortality was observed after 16 Gy PBI. The survival results were then re-plotted as mortality percentage against each irradiation dose (Fig. 5b). We have utilized Probit(P) analysis using Microsoft Excel to determine the LD_50/30_. Using the most linear part of the mortality curve, i.e., 13.5, 14 and 14.5 Gy, the DMF was calculated as 1.058 (Fig. 5c). Thus, the treatment increased the radiation dose needed to kill 50% of the mice by 1 Gy, which is a critical benchmark requirement for candidate GI-ARS MCMs^27^.

### Human cell-expressed tag-free rhMFG-E8 restored plasma citrulline levels and improved gut histology after PBI

**Figure 6:**
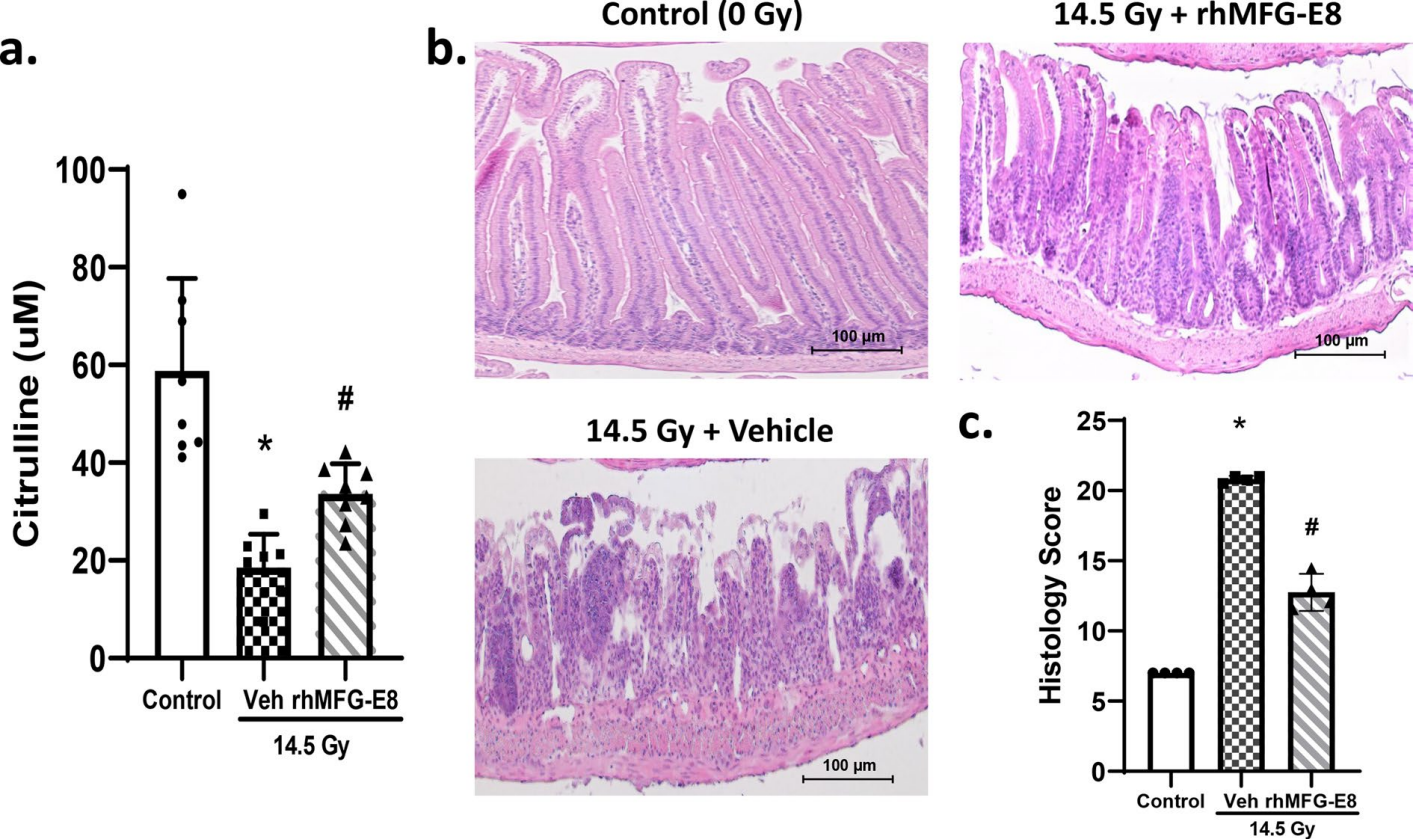
Tag-free rhMFG-E8 increased plasma citrulline and improved gut histology in PBI mice**.** (**a**) Mice were exposed to PBI and injected sc with normal saline (14.5 Gy + Vehicle) or 1 µg/kg tag-free rhMFG-E8 (14.5 Gy + rhMFG-E8) starting at 24 h post-PBI and blood samples were collected at 3-days after PBI. Serum levels of citrulline were measured by Mouse Citrulline ELISA Kit. Mice that were not exposed to irradiation was used as control (0 Gy) group. (**b**) Representative images of gut histological samples from Day-3 after PBI are shown. (**c**) The histological damage score quantified using RIIMS are shown. Data are represented as mean ± SE (n = 8/group) and analyzed using one way ANOVA and Student Newman-Keuls test. *P < 0.05 versus control; ^#^P < 0.05 versus Vehicle.

Citrulline is a nitrogen end-product of glutamine metabolism in small-bowel enterocytes, and plasma citrulline has been identified as a biomarker of radiation-induced gut damage^26^. To assess gut damage, mice were subjected to 14.5 Gy PBI and at 24 h later, they were treated daily with 1 µg/kg BW tag-free rhMFG-E8. Our data showed that the serum levels of citrulline were decreased in vehicle group as early as 3-days after PBI whereas tag-free rhMFG-E8 treatment group showed a significant increase in citrulline levels (Fig. 6a). These results indicate that improved enterocyte activity could be detected as early as after only two administrations of tag-free rhMFG-E8. We also assessed rhMFG-E8’s effects on the intestinal histological architecture. Regenerative crypts appeared very early following radiation injury in treated mice. Treatment with tag-free rhMFG-E8 in PBI mice attenuated histological damage and further confirmed the protective effect of tag-free rhMFG-E8 on radiation-induced gut damage (Fig. 6b). We further quantified the histological changes using RIIMS and showed that tag-free rhMFG-E8 treatment significantly attenuated gut damage (Fig. 6c). These results demonstrated tag-free rhMFG-E8’s efficacy in vivo, and support further developing it as a biological MCM for GI-ARS.

## Discussion

MFG-E8, also known as lactadherin in humans, is a glycoprotein secreted by activated phagocytes that bridges the phosphatidylserine (PS) residues on the surface of dying (mostly apoptotic, but also autophagic, necrotic, necroptotic, pyroptotic) cells with α_V_β_3_ and α_V_β_5_ integrins on the surface of macrophages and dendritic cells^4,5,28,29^. MFG-E8 promotes the uptake and clearance of dying cells and prevents local and systemic immune activation and end-organ damage during sepsis, ischemia/reperfusion injury and shock. We have previously demonstrated the beneficial effect of bacterially expressed His-tagged rhMFG-E8 in various organ injury conditions^11–15,30^. However, unlike eukaryotic expression systems, *E. coli* lack the ability to produce complete and proper post-translational modifications such as glycan glycosylation, which are important for the adequate protein folding and biological activity of glycoproteins such as MFG-E8. Moreover, *E. coli* expressed proteins run the risk of carry-over of endotoxin and other toxic bacterial components. While the presence of a His-tag facilitates recombinant protein recovery and purification, at the same time it can render proteins immunogenic and thus unsuitable for drug development. To circumvent these undesirable effects, we used a human cell expression system (HEK293 cells) to produce tag-free rhMFG-E8, which we then purified, authenticated, quality-controlled for biological activity in vitro, and demonstrated to be effective in vivo using preclinical models of human disease.

The biological integrity of the tag-free rhMFG-E8 was examined in vitro by a PS binding assay, which we have developed and standardized in house, as well as by the classical cell adhesion assay. It was evident from its steep linear phase slope that the tag-free rhMFG-E8 was significantly better than *E. coli*-expressed His-tagged rMFG-E8 and had comparable PS binding activity to also mammalian cell-expressed but His-tagged commercially available rhMFG-E8. Tag-free rhMFG-E8 also bound significantly more strongly to SVEC4-10 cells than *E. coli*-expressed His-tagged rhMFG-E8, and equally or more strongly than commercial rhMFG-E8 in all different dilutions, indicating strong ability of the tag-free rhMFG-E8 to bind to the integrin receptors α_V_β_3_ and α_V_β_5_. These results mean that tag-free rhMFG-E8 can be lyophilized into powder form, stored for long periods of time, and reconstituted all the while preserving its full biological function. Moreover, mice administered up to 2 mg/kg BW tag-free rhMFG-E8 (2000-fold the highest therapeutic dose in our study) showed no detectable toxicity or inflammation and no significant changes in body weight for up to 28 days after the administration of tag-free rhMFG-E8, indicating lack of toxicity. Nevertheless, additional experiments are required for assessing longer term safety. Additionally, tag-free rhMFG-E8 protein had a relatively short half-life that is adequate for commonly used drug administration regimens, and suggest it is very unlikely that rhMFG-E8 will accumulate in the body to cause adverse effects. Finally, the endotoxin level in tag-free rhMFG-E8 was substantially lower than the industry standard. Together, these observations clearly demonstrate the biological activity, safety, and stability of the tag-free rhMFG-E8 in vitro.

To evaluate the biological activity of the human cell-expressed tag-free rhMFG-E8 in vivo, an experimental mouse model of PBI was utilized. PBI is accomplished by sparing the bone marrow either extensively (40%) or minimally (5%). Our study was conducted using the 5% bone marrow sparing model. This model was chosen because it is an established model in primates to assess MCM effects on the major radiation syndromes (H-ARS, GI-ARS, DEARE)^27^. In the current study of 15 Gy PBI, survival rate significantly improved from 25% in the vehicle to 89% with 1 µg/kg BW treatment for 30-days demonstrating the efficiency of the tag-free rhMFG-E8 in ameliorating PBI-induced ARS. Animal survival after TBI can increase from 37 to 87% with a small increment in the radiation dose^31^. Indeed, we observed 100% survival with 13 Gy and 100% mortality with 15 Gy in our PBI model. Therefore, the effectiveness of the 1 µg/kg BW tag-free rhMFG-E8 was assessed using full dose response curves from 13 to 16 Gy in increments of 0.5 Gy. We measured effectiveness using the standardized method of DMF, i.e., the radiation dose required for a given effect (in our case, for LD_50/30_) in treated animals divided by the radiation dose leading to the same effect in the control group^27^. Treatment with tag-free rhMFG-E8 caused a 1 Gy shift in the radiation LD_50/30_ thus fulfilling part of the FDA’s “animal rule” efficacy requirement to approve candidate MCMs for GI-ARS^27^.

Another criterium for assessing a candidate MCM is that it should ameliorate the intestinal abnormalities characteristic of GI-ARS such as epithelial denudation and crypt loss. Thus, in mice irradiated with PBI, we also assessed the beneficial effects of tag-free rhMFG-E8 on the plasma levels of citrulline and the histological architecture of the small intestine. Citrulline is an epithelial tissue-specific nitrogen end product of glutamine metabolism in small-bowel enterocytes. Since a lower plasma concentration of citrulline correlates with decreased enterocyte mass/integrity, its circulating levels have been used as a GI-ARS severity biomarker^26^. After PBI, we observed a significant decrease in citrulline levels and intestinal histological injury in the vehicle group, but treatment with tag-free rhMFG-E8 significantly ameliorated enterocyte mass and function, as indicated by the partially restored citrulline levels. Control mice subjected to PBI also had loss of intestinal crypts and breakdown of the mucosal barrier, with sloughing of the tips of the villi and denudation of the epithelial cell layer. On the other hand, mice treated with tag-free rhMFG-E8 had significantly lower intestinal histological injury scores. Breakdown of the intestinal mucosal barrier causes electrolyte imbalances and bacterial translocation (including their toxins) through the intestinal wall into the bloodstream, thus predisposing to infection and inflammation. It is pertinent to also note the paucity of abnormal mitotic nuclei in the crypt after treatment with tag-free rhMFG-E8. This positive effect of tag-free rhMFG-E8 on the gut after PBI can also be attributed, at least in part, to MFG-E8’s trophic effects leading to the repair of the damaged intestinal epithelium and preservation of gut homeostasis^4,32^. It would have been interesting to examine gut injury under different PBI dose. However, a separate experiment with the 14.5 Gy dose was used to generate the gut injury data. Additional experiments are indeed needed to examine gut injury in the remaining PBI doses used in the study. Future studies are warranted for such analysis.

We estimated the sample size for the survival study with the expectation that rhMFG-E8 treatment will increase the survival by ~ 50%. Thus, the estimated sample size based on proportion was 20 mice per group. The original survival study included 20 mice per group where we observed 25% survival in the vehicle group and 89% survival with 1 ug/kg rhMFG-E8 treatment indicating a 64% increase suggesting 20 mice per group was sufficient to obtain adequate power for the study. Except for the 15 Gy group, lesser than 20 mice were used to generate the DMF curve. Among all the PBI doses analyzed, statistically significant difference between vehicle and rhMFG-E8 treatment was seen with 14.5 Gy and 15 Gy. We did not achieve statistical significance between groups for the remaining radiation doses used perhaps due to lesser than 20 mice used for those doses. Future studies are warranted for such conclusion.

In our study, we chose to employ adult C57BL/6 J mice because they are the most widely studied strain and species in preclinical studies of radiation injury^27^. Furthermore, C57BL/6 mice exhibit a moderate degree of radiosensitivity compared to other strains^33^. Some studies indicated that radiosensitivity depends on the timing of irradiation^34^ and that some anesthetics can act as radiation protectants^35^. Therefore, all mice were non-anesthetized and X-Ray irradiated in the morning with a single exposure at a rate of 1 Gy/min. Since drift in the LD_50/30_ occurs within any laboratory, it is recommended that every laboratory establish a lethality dose–response for each mouse strain at least twice every year^27^. Accordingly, we first exposed different doses of X-ray using PBI model to determine the proper dose in C57BL/6 J mice to be used for rhMFG-E8 treatment studies. Our initial experiment determined the LD_50/30_ as 15 Gy, which we then selected to determine the dose response relationship and the optimal dose of the tag-free rhMFG-E8 in treating PBI mice. However, when conducting the dose modification factor studies, 15 Gy had 100% mortality and the LD_50/30_ shifted to 13.7 Gy. We believe this discrepancy is due to the inherent drift that naturally occurs over time as well as the subtle batch to batch variation in mice^27^. In anticipation for such variation, all experiments included contemporaneous vehicle (saline) injection controls. Treatment with 1 µg/kg BW tag-free rhMFG-E8 increased the LD_50/30_ by 1 Gy, strongly supporting further evaluating tag-free rhMFG-E8 as a radiation MCM in large animal models such as dogs or non-human primates.

We administered tag-free rhMFG-E8 by subcutaneous injection, as opposed to the intravenous route as chosen for the PK study. One can question that shouldn’t the method consistency be maintained throughout the study. Since the objective of our study was to develop rhMFG-E8 as a radiation mitigator for clinical scenario such as a nuclear disaster or terrorism, we have chosen subcutaneous route to accommodate the feasibility in treatment for mass casualties encountered by medical and paramedical personnel. Intravenous administration would require specialized training which could limit the efficiency of the treatment. Furthermore, PBI mice were treated 24 h after radiation exposure, once again reflecting a mass casualty scenario when a large number of victims may overwhelm the system and preclude immediate care. However, we recognize that the difference in route of administration could show differences in the pharmacokinetic properties of the compound and future PK studies should be conducted using subcutaneous route of administration. Therefore, we consider the discrepancies in the route of administration as a limitation of the study.

We and others have previously shown that MFG-E8 functions as an anti-inflammatory factor which has been involved in multiple biological processes including innate immunity^5,9^. In fact, decrease in MFG-E8 expression has been correlated with coronary artery atherosclerosis^36^. Circulating levels of MFG-E8 were significantly declined in failing hearts from patients with dilated cardiomyopathy. Deficiency in MFG-E8 exacerbated cardiac hypertrophy whereas administration of human recombinant MFG-E8 in normal mice alleviated aortic banding induced cardiac hypertrophy. This study clearly suggests that MFG-E8 is an endogenous negative regulator of pathological cardiac hypertrophy indicating MFG-E8 as a therapeutic target for treatment of cardiac hypertrophy^37^. However, MFG-E8 may play a proinflammatory role, depending upon the contexts such as cells, organs, and age. For instance, MFG-E8 promotes inflammatory vascular remodeling with advancing age and tumor progression, including breast cancer and melanoma^38–40^. MFG-E8 is expressed in aged arteries and thus has been identified as a novel biomarker for arterial aging^41,42^. Since MFG-E8 can be induced by angiotensin II peptide and that treatment of MFG-E8 with VSMCs stimulate VSMC proliferation and invasion suggests a proinflammatory role of MFG-E8 in aging arteries. Subsequently, another study reported that MFG-E8 promoted the proinflammatory phenotype in aged vascular smooth muscles (VSMCs) and arteries suggesting treatment with exogenous MFG-E8 renders the vasculature prone to vascular diseases^43^. In addition, during aging, MFG-E8 could be cleaved into small 50-aa peptide named medin, which is a potent inflammatory molecule and very common amyloid protein observed in the old aortic, temporal, and cerebrovascular walls. However, it is still not known what, when, and how local proteinases cleaves MFG-E8 into medin in the arterial wall^44^. To explore the proinflammatory role of MFG-E8 in radiation mitigation, further studies are warranted in the future.

It would have been interesting to know whether MFG-E8 reduces the hematopoietic acute radiation syndrome induced by high PBI dosage. In this regard, we have previously measured hematopoietic parameters such as white blood cell counts, red blood cell count, hemoglobin, hematocrit, and platelet count at 20 h and one week after whole body irradiation in rats. With exception of the white blood cell count, all measurements were similar to sham levels. The white blood cell count dramatically decreased as early as 20 h and the treatment with His-tagged rhMFG-E8 slightly improved the count but the data were not significant^11^. These findings will not exclude the possibility that treatment with rhMFG-E8 after irradiation could be protective of the hematopoietic systems. Future studies are warranted for such conclusion.

MFG-E8 was identified as a surface protein of mammary epithelial cells but its physiological role had not been known for a long time. In 2002, Hanayama et al., found that MFG-E8 secreted by macrophages and immature dendritic cells acts as a bridging molecule between apoptotic cells and phagocytes^5,45^. MFG-E8 has a high affinity to PS which are exposed on apoptotic cells but not with other phospholipids on the cell membrane. While engaged with apoptotic cells, MFG-E8 interacts with phagocytes to stimulate the uptake of apoptotic cells. MFG-E8 contains an RGD motif in the second EGF domain which is a ligand for α_V_β_3_ or α_V_β_5_ integrin expressed on various phagocytes including amateur and professional phagocytes. Although the role of these integrins in apoptotic clearance had been well established, neither of these integrins bind directly to PS. Being able to bind both PS on apoptotic cells and integrins on phagocytes MFG-E8 acts as an opsonin for the uptake of apoptotic cells. MFG-E8 promotes the phagocytosis of apoptotic cells by not only MFG-E8 secreting macrophages but also by other phagocytes. MFG-E8 deficient mice develop inflammation and autoimmunity, including glomerulonephritis, attributed to defects in apoptotic cell removal^7^.

The molecular and cellular mechanisms behind mitigating radiation induced injuries in the gut have not been completely elucidated. A possible mechanism by which rhMFG-E8 confers therapeutic advantage after radiation is upregulating p53, a tumor suppressor protein that acts as a regulator of the cell cycle. In normal cells, low levels of p53 are maintained as p53 binds to MDM2 and other negative regulators. After stress condition, p53 levels accumulate in the cell through the inhibition of its interaction with the negative regulators^46–48^. Deleting p53 from the gastrointestinal tract (GI) epithelium sensitized irradiated mice to the GI syndrome and that overexpression of p53 were protected from the GI syndrome after irradiation^49,50^. We have previously shown that treatment with rhMFG-E8 increased p53 expression in the ileum of rats subjected to whole body irradiation^11^. By increasing p53, a major regulator of the cell cycle, rhMFG-E8 improves cell survival and protects the gut epithelium. Secondly, we have shown that Bcl-2, an anti-apoptotic marker, was significantly increased in the ileum of rhMFG-E8 treated rats while its expression was decreased in the vehicle group^11^. However, future studies are required to pinpoint the exact mechanism in preserving gut integrity by rhMFG-E8 after radiation injury.

In addition to tag-free rhMFG-E8’s potential to be developed as a biological therapeutic for radiation injury, it might also be developed for other diseases such as atrial fibrosis and atrial fibrillation^51^, subarachnoid hemorrhage^52,53^, osteoarthritis^54^, and acute pancreatitis^55^. Additionally, MFG-E8 has the potential to become the first example of an entirely new biologic able to promote health and healing by enhancing the removal of dying cells in situations characterized by cytotoxicity, such as infectious diseases, intoxications, envenomations, and in patients undergoing chemotherapy and radiotherapy.

## Conclusions

In summary, we have successfully generated human cell-expressed tag-free rhMFG-E8 with elevated and stable biological activity and strong safety and pharmacokinetic profiles well suited for the development of therapeutics in acute inflammation and injury. Tag-free rhMFG-E8 effectively ameliorated experimental models of radiation injury. Therefore, human cell-expressed tag-free rhMFG-E8 is a strong candidate to become an effective radiomitigator.

### Supplementary Information

Supplementary Figure 1.

Supplementary Figure 1.

Supplementary Figure 1.

## Data Availability

All data generated or analyzed during this study are included in this published article [and its Supplementary Information files].

## Abbreviations

rhMFG-E8: Recombinant human milk fat globule-epidermal grow factor-factor
PBI: Partial body irradiation
DMF: Dose modification factor
EGF: Epidermal growth factor
ARS: Acute radiation syndrome
H-ARS: Hematopoietic acute radiation syndrome
GI-ARS: Gastrointestinal acute radiation syndrome
NV-ARS: Neurovascular acute radiation syndrome
MCM: Medical countermeasure
TBI: Total body irradiation
BUN: Blood urea nitrogen
PS: Phosphatidylserine
PBS: Phosphate buffered saline
HRP: Horseradish peroxidase
TMB: 3,3',5,5'-Tetramethylbenzidine
ELISA: Enzyme linked immunosorbent assay
TUNEL: Terminal deoxynucleotidyl transferase dUTP nick end labeling
Gy: Gray
LD: Lethal dose
