# Supplementary figures and images for: Human cell-expressed tag-free rhMFG-E8 as an effective radiation mitigator
Source: Sci Rep. 2023 Dec 13;13:22186. doi: 10.1038/s41598-023-49499-y (PMC10719321; doi:10.1038/s41598-023-49499-y)

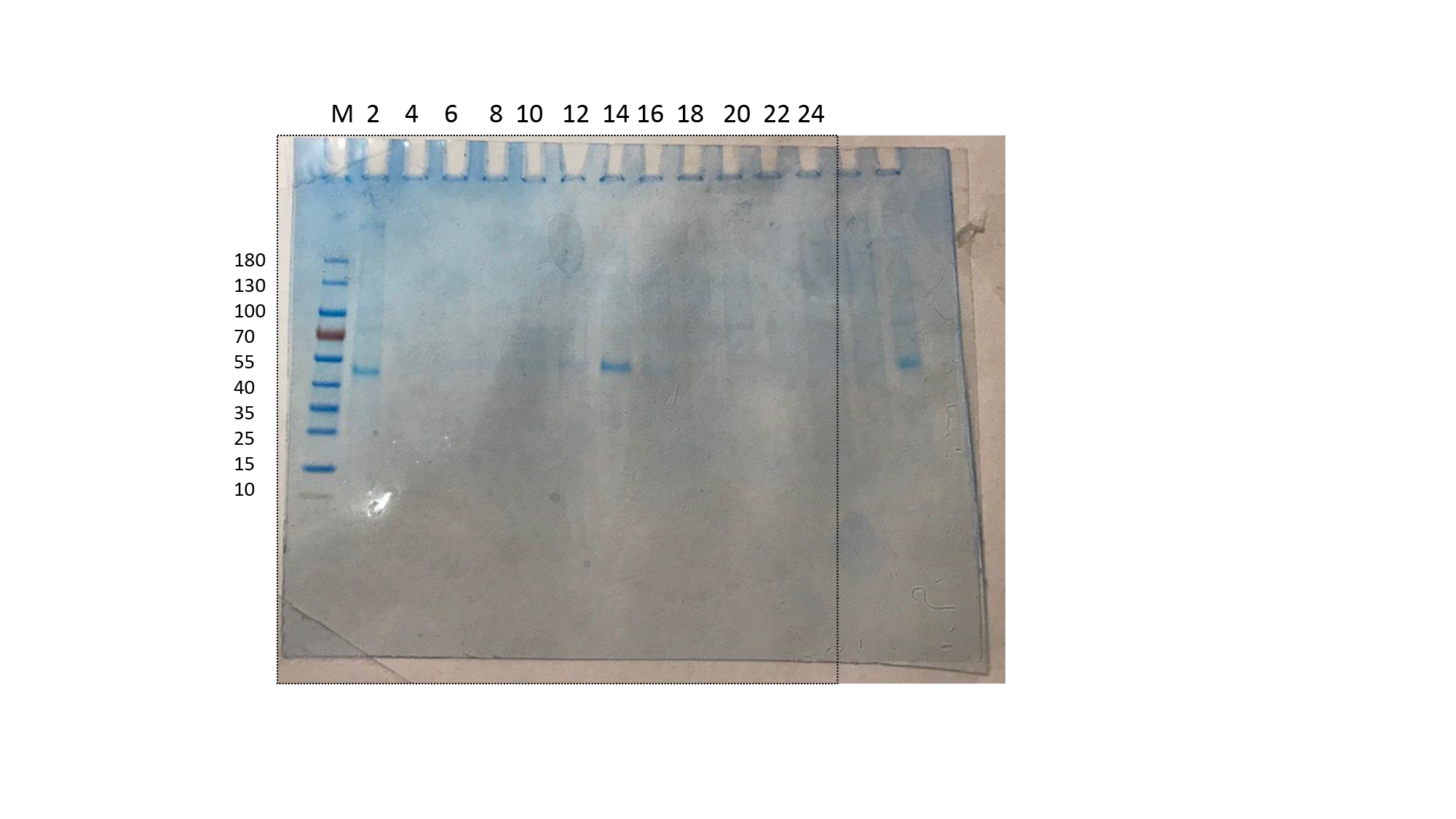

Supplement: Supplementary file 1 — Supplementary Figure 1. [file 41598_2023_49499_MOESM1_ESM.tif]

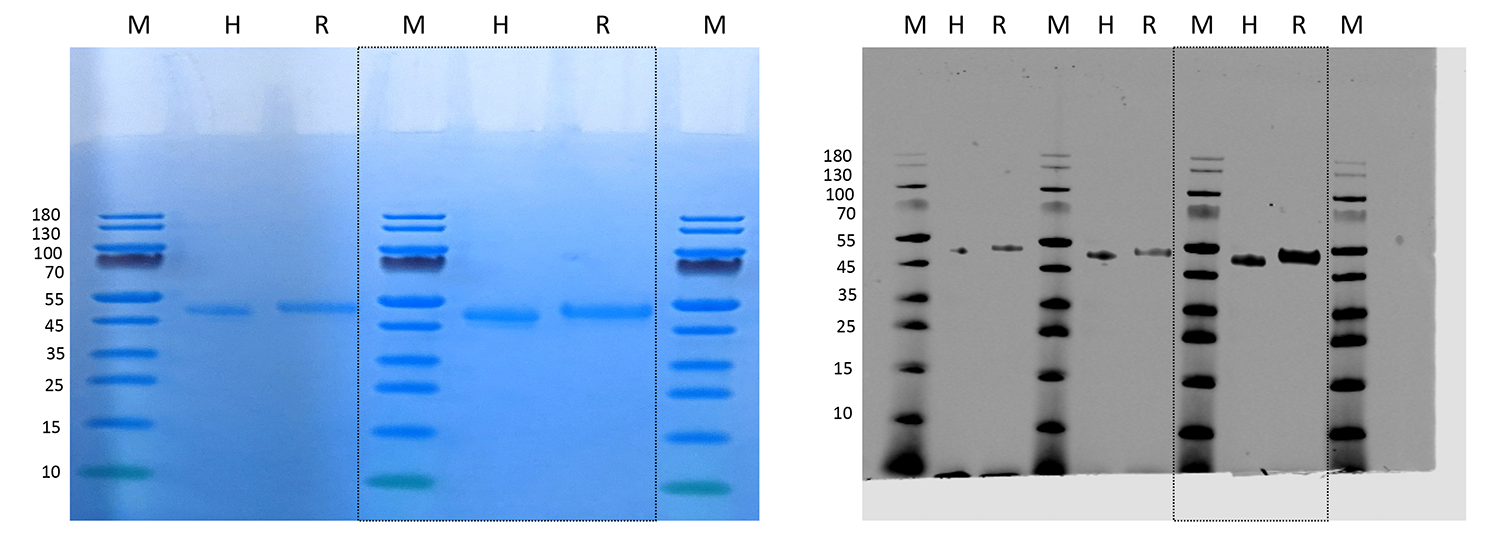

Supplement: Supplementary file 2 — Supplementary Figure 1. [file 41598_2023_49499_MOESM2_ESM.tif]

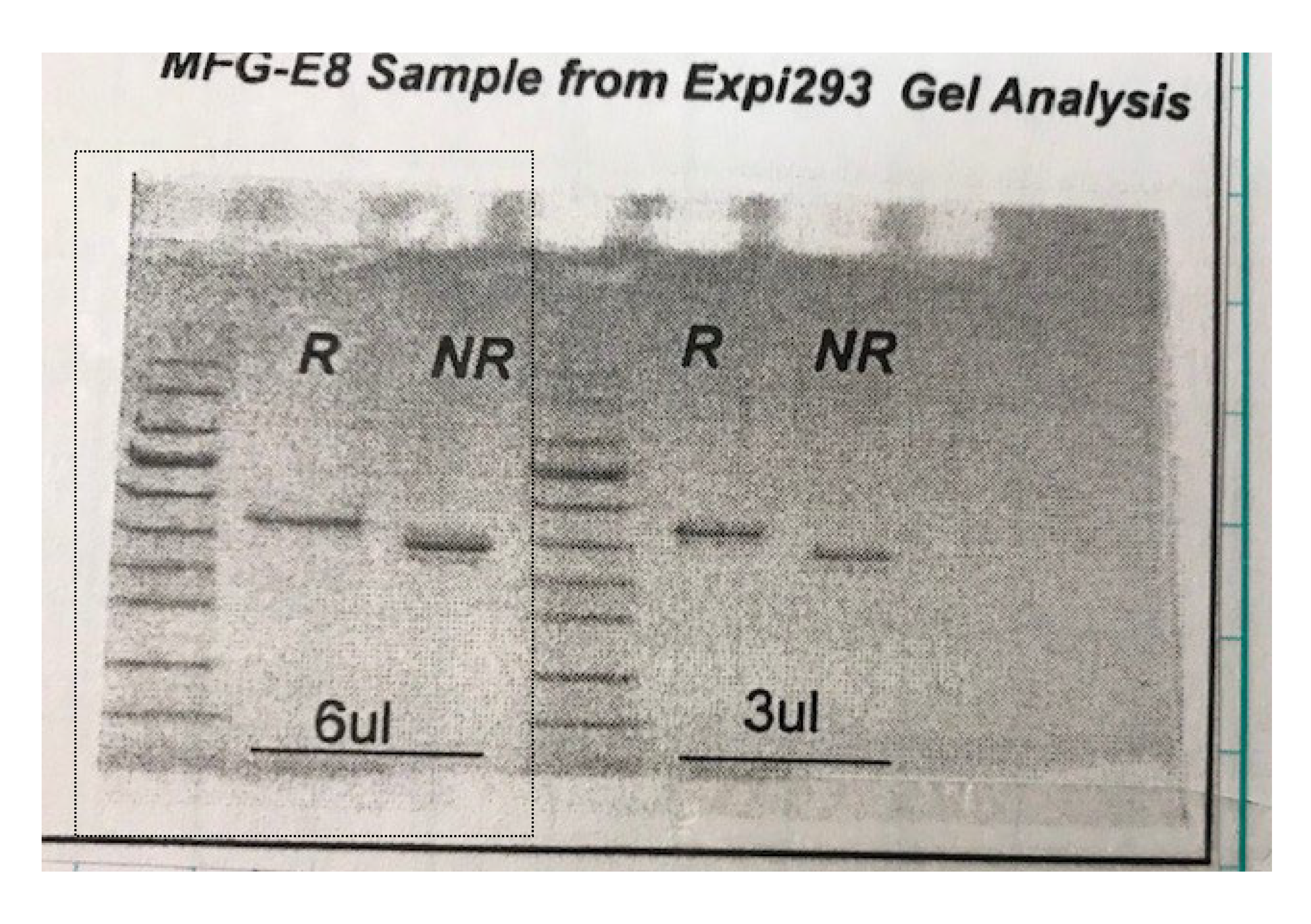

Supplement: Supplementary file 3 — Supplementary Figure 1. [file 41598_2023_49499_MOESM3_ESM.tif]
